# Commentary: The sphingosine kinase 1/sphingosine-1-phosphate pathway in pulmonary arterial hypertension

**DOI:** 10.3389/fphar.2015.00229

**Published:** 2015-10-19

**Authors:** Naga P. Bhavanam, Athena Failla, Young Cho, Richard F. Lockey, Narasaiah Kolliputi

**Affiliations:** Department of Internal Medicine, Morsani College of Medicine, University of South FloridaTampa, FL, USA

**Keywords:** pulmonary arterial hypertension, hypoxia, sphingosine kinase 1, sphingosine-1-phosphate, pathway

Pulmonary arterial hypertension (PAH) is a progressive and irreversible lung disease that reduces survival. Importantly, the pulmonary arteries carry blood from the heart to the lungs; however, in PAH pulmonary vessels are remodeled causing abnormal constriction and a pressure change that prevents an adequate supply of oxygen delivered to the lung tissue (Farber and Loscalzo, 2004). PAH is categorized as a pulmonary artery pressure greater than 25 mmHg, with various factors contributing to the pathogenesis of the disease, including higher levels of endothelin-1, decreased nitric oxide (NO) synthase expression, and inhibition of the prostacyclin pathway which leads to decreased relaxation and induced proliferation of pulmonary artery smooth muscle cells (PASMCs) (McLaughlin and McGoon, 2006). Inflammation, toxins, lack of oxygen, and the deregulation of coagulation factors, are associated with endothelial cell dysfunction in PAH (McLaughlin and McGoon, 2006). The main pathologic changes associated with PAH include vasoconstriction, endothelial and smooth-muscle cell proliferation, thrombosis, and pulmonary remodeling (Farber and Loscalzo, 2004). PAH may eventually cause an enlarged right ventricle and compressed left side of the heart, leading to right ventricular heart failure in severe cases (McLaughlin and McGoon, 2006). It has also been theorized that glycolysis and oxidative phosphorylation play a role in patients with PAH, mediating cell proliferation, migration, and angiogenesis (Chen et al., 2014). Individuals afflicted with PAH have dyspnea, angina, fatigue, and increased pulmonary resistance (McLaughlin and McGoon, 2006). Without medical treatment, the survival rate is estimated to be less than 3 years after diagnosis (Cottrill and Chan, 2013). Thus, PAH is a serious illness and can be fatal, which signifies the importance of finding novel targets that can control and regulate this disease (Beckham et al., 2013; Chen et al., 2014).

Although current treatment options for PAH include phosphodiesterase-5 inhibitors, prostanoids, calcium channel blockers, endothelin receptor antagonists, diuretics, oxygen therapy, and surgical procedures, such as atrial septostomy, these treatments only delay the progression of the disease (McLaughlin and McGoon, 2006; Tuder et al., 2012). Thus, a cure for PAH remains to be elucidated. Interestingly, recent research suggests that two important kinases, sphingosine kinase 1 (SphK1) and sphingosine kinase 2 (SphK2), are primary therapeutic targets for pulmonary hypertension (Cottrill and Chan, 2013; Chen et al., 2014). It is reported that SphK1 is a promoter for tumor progression, invasion, and metastasis in gastric, prostate, colon, breast, and small cell lung cancers (Beckham et al., 2013; Yang et al., 2014). Overexpression of SphK1 is known to stimulate the production of various cell types, such as NIH 3T3 fibroblasts, intestinal epithelial cells, and endothelial cells (Chen et al., 2014). A study by Chen and his colleagues showed that SphK1 and SphK2 regulate phosphorylation, and therefore synthesis of sphingosine-1-phosphate (S1P), a bioactive sphingolipid that mediates the lipid bilayer and promotes cell proliferation, migration, and angiogenesis (Chen et al., 2014). Additionally, SphK1 is the most prevalent form of the two kinases and is found in the lungs, kidneys, blood and spleen (Chen et al., 2014).

In a recent study, Chen et al. (2014) provides experimental evidence suggesting that SphK1/S1P signaling may be a promising therapeutic pathway to treat PAH (Figure 1). Chen et al. (2014) investigated the pathway in two experimental models: mice and rats (rodents) with hypoxia-induced PH and patients with idiopathic PAH. The results show that expression levels of SphK1 and S1P were considerably higher in the lungs of patients with PAH and in PH rodent models after exposure to hypoxia (10% O_2_) for 4 weeks, compared to normoxic control animals. Immunoblot analysis validated elevated SphK1 and SphK2 protein levels, with β-actin acting as a control to indicate the increase in activity of both proteins. However, SphK2 expression was not changed when compared to the normoxic controls. SphK1 and S1P levels, measured by C18-S1P, were also elevated in the lung tissue and pulmonary arteries isolated from rodent models. Inhibition of SphK1 reduced cell proliferation and pathological indications of vascular remodeling *in vivo*, suggesting the role of SphK1 in causing pulmonary hypertension.

**Figure 1:**
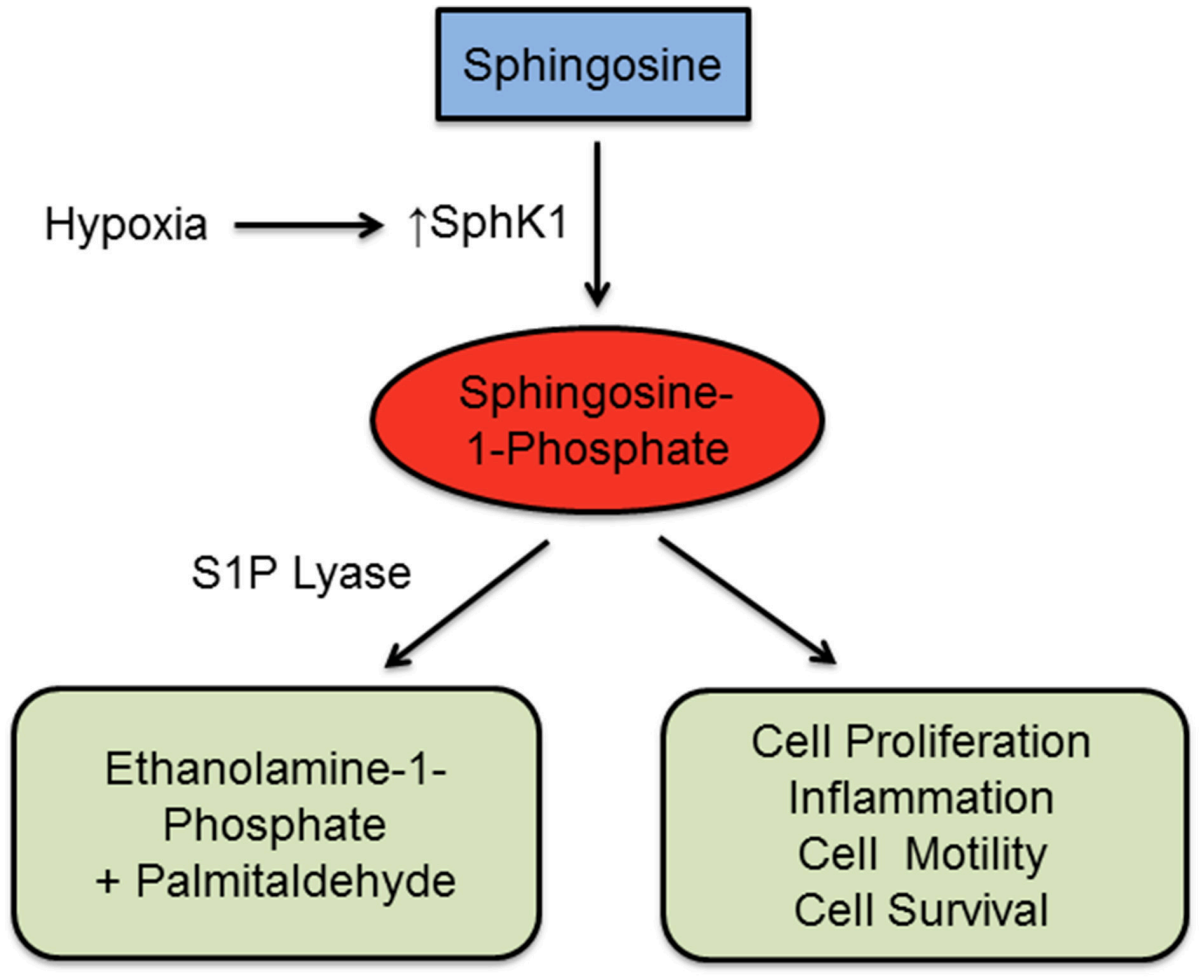
**Mechanistic pathway of SphK1 and S1P**. Exposure to hypoxia induces an upregulation of SphK1, which phosphorylates sphingosine and produces spingosine-1-phosphate (S1P). Higher concentrations of S1P will induce cell proliferation, tissue inflammation, cell motility, and cell survival. These responses promote the progression of pulmonary arterial hypertension (PAH). S1P can also be cleaved by S1P lyase, generating the stable non-sphingolipid products ethanolamine-1-phosphate and palmitaldehyde.

Chen et al. (2014) also compared S1P lyase (*Sgpl*1)-deficient mice exposed to hypoxia to wild-type *Sgp*1 mice to examine whether increased levels of S1P contributes to the pathogenesis of PH. *Sgpl*1-deficient mice showed increased expression of S1P in both normoxia and hypoxia conditions. After a 6-week hypoxia exposure, elevated right ventricular systolic pressure (RVSP) and pulmonary vascular remodeling developed in *Sgpl*1-deficient mice; however, these outcomes were not observed in wild-type mice. Furthermore, Chen et al. (2014) showed that the SphK1/S1P pathway is dependent on ligation to the sphinogosine-1-phosphate receptor 2 (S1PR2) in human pulmonary arterial smooth muscle cells (PASMCs), after expression of S1PR2 was confirmed. Inhibition of S1PR2 by JTE013 antagonist further confirmed the ligation process of the SphK1/S1P pathway.

Chen et al. (2014) demonstrated that uncontrolled PASMC cell reproduction and its resistance to apoptosis are main components of the PAH mechanistic pathway. Additionally, the results show that S1P promoted PASMC cell proliferation is dose-dependent and plays a major role in pulmonary vascular remodeling evident in PAH. Taken together, Chen et al. (2014) and his colleagues produced significant evidence to show that Sphk1 is associated with the pathophysiology of PAH. These findings are significant because they provide a foundation on which to base further research in order to treat patients with PAH. Experimental evidence demonstrates that at the cellular level, PAH and hypoxia-induced PH is caused by multiple factors (Cottrill and Chan, 2013). Chen et al. (2014) also demonstrated that S1P regulates PASMC proliferation and regulates the expression of proapoptotic and antiapoptotic proteins (Chen et al., 2014). Consequently, altering the metabolic pathway of SphK1/S1P may serve as an alternative approach to ameliorate the progression of PAH (Chalfant and Spiegel, 2005; Tuder et al., 2012; Beckham et al., 2013). These results indicate that the SphkK1/S1P pathway promotes PASMC cell proliferation and pulmonary remodeling, suggesting that SphK1, S1P, and S1PR2 are future therapeutic targets for the treatment of PAH.

## Conflict of interest statement

The authors declare that the research was conducted in the absence of any commercial or financial relationships that could be construed as a potential conflict of interest.
